# EXPRESSION OF CONCERN: KLF4 interacts with TXNIP to modulate the pyroptosis in ulcerative colitis via regulating NLRP3 signaling

**DOI:** 10.1002/iid3.1199

**Published:** 2024-02-27

**Authors:** Yuan Chen, Lifeng Sun, Haiyan Liu, Jiamei Li, Lu Guo, Zhiyi Wang

**Affiliations:** ^1^ Department of Pediatrics Shandong Provincial Hospital Affiliated to Shandong First Medical University Jinan Shandong People's Republic of China; ^2^ Shandong Provincial Clinical Research Center for Children's Health and Disease Office Shandong Provincial Hospital Affiliated to Shandong First Medical University Jinan Shandong People's Republic of China; ^3^ Department of Pathology Shandong Provincial Hospital Affiliated to Shandong First Medical University Jinan Shandong People's Republic of China; ^4^ Department of Hepatobiliary Surgery Shandong Provincial Hospital Affiliated to Shandong First Medical University Jinan Shandong People's Republic of China

**Keywords:** ATP/LPS, KLF4, NLRP3, TXNIP, ulcerative colitis

## Abstract

**Introduction:**

Ulcerative colitis (UC) is one of the most common diseases in the gastrointestinal tract related to abnormal inflammation. Pyroptosis, which is characterized by the formation of inflammasome, activation of caspase‐1, and separation of N‐ and C‐terminus of gasdermin D (GSDMD), and may be involved in the pathogenesis of IBD. Krüppel‐like factor 4 (KLF4) is a zinc finger transcription factor expressed in differentiated epithelial cells. KLF4 mediates proinflammatory signaling in macrophages. Here, we tested whether KLF4 is functional in pyroptosis of UC.

**Methods:**

In human UC tissues and/or lipopolysaccharide (LPS)/adenosine 5‐triphosphate (ATP) stimulation human colon epithelial cells, KLF4, TXNIP, Cleave‐Caspase‐1, and GSDMD expression were detected through quantitative reverse transcription polymerase chain reaction (PCR), immunohistochemical and western blot assay. Interleukin (IL)‐1β and IL‐18 levels were quantified by enzyme‐linked immunosorbent assay. We successfully constructed a KLF4‐silenced colon epithelial cell line using an adenovirus vector. We apply the UCSC and JASPAR to predict the KLF4 binding sites in the promoter region of TXNIP.

**Results:**

In human UC tissues and/or LPS/ATP stimulation human colon epithelial cells, KLF4, TXNIP, Caspase‐1, and GSDMD expression level were significantly elevated via quantitative reverse transcription PCR, immunohistochemical and western blot assay. Moreover, We identified that there is an interaction between KLF4 and TXNIP through Yeast double hybrid assay and CO‐IP assay. We successfully constructed a KLF4‐silenced human intestinal epithelial cell line. In LPS/ATP stimulation KLF4‐silenced human intestinal epithelial cells, KLF4, TXNIP, Cleave Caspase‐1, ASC, and GSDMD expression level were significantly decreased via quantitative reverse transcription PCR.

**Conclusion:**

Our results confirm that KLF4 can positively regulate the expression of TXNIP and regulate the pyroptosis process of UC through the TXNIP/NLRP3 pathway.

## Graphical abstract

Our results confirm that Krüppel‐like factor 4 can positively regulate the expression of TXNIP and regulate the pyroptosis process of ulcerative colitis through the TXNIP/NLRP3 pathway.

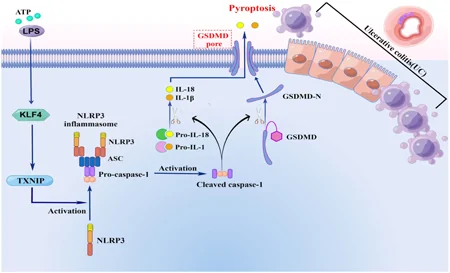

## 1 INTRODUCTION

Ulcerative colitis (UC) is a chronic nonspecific inflammatory disease of gastrointestinal tract with unknown etiology. In recent years, the incidence of UC has been on the rise and has become one of the major diseases threatening public health worldwide.^1^, ^2^ UC has a long course of disease, frequent recurrence, difficult cure, and susceptibility to cancer, which greatly increases the burden on families and society, and also brings great challenges to the medical and health industry.^2^, ^3^ However, its etiology and pathogenesis have not been fully elucidated.

Cell pyroptosis includes classical pathway and nonclassical pathway, and the nonclassical pathway is mainly initiated by human Caspase‐4, human Caspase‐5, or mouse Caspase‐11. The classical pathway is mainly dependent on Caspase‐1 induction, and the formation of NLRP3 inflammasome is an important step. Cytoplasmic sensing proteins of different pathogens and inflammatory factors trigger NLRP3 proteins to form the NLRP3‐ASC‐pro‐Caspase‐1 complex, also known as the NLRP3 inflammasome.^4^ Procaspase‐1 is Cleaved to active Caspase‐1 (Cleaved caspase‐1), cleaved caspase‐1 promotes the cleaved and mature of interleukin‐1β (IL‐1β) and interleukin‐18 (IL‐18) preforms. Moreover, gasdermin D (GSDMD) was cut to form cytotoxic GSDMD‐N, which was recruited to the cell membrane to form GSDMD‐N cell pores, resulting in cell swelling, membrane rupture, and overflow of cell contents, leading to cell pyrosis.^5^, ^6^, ^7^ At the same time, mature IL‐18 and IL‐1β can be released into the extracellular through GSDMD‐N cell pores, recruiting inflammatory cells to gather and eventually lead to inflammatory storms in the body.

During the onset of inflammatory activity in UC, intestinal epithelial cells can undergo a variety of cell death pathways, including apoptosis, necrosis, necrotic apoptosis, pyroptosis, and ferroptosis.^8^ However, there are still gaps in the basic knowledge of the mechanism of UC intestinal epithelial cell death. In summary, there is an urgent need for a deeper understanding of the mechanisms underlying UC intestinal epithelial cell death to develop new and promising therapeutic strategies.

Krüppel‐like factor 4 (KLF4) is a eukaryotic zinc finger transcription factor with specific binding sites. It is a member of the KLF‐like factor family and belongs to the C2H2 type zinc finger protein. Due to its abundant expression in the digestive system, endothelial cells, and skin, it is also known as a gastrointestinal enriched Krüppcl‐like factor. The KLF4 protein has multiple functional domains with highly conserved carboxyl‐terminal sequences, including three C2H2 zinc finger structures containing 81 amino acids, which are DNA binding domains. The amino terminal of KLF4 protein contains a highly variable transcriptional regulatory domain, the transcriptional activation domain (91–117 amino acid residues) is rich in proline and serine, and the transcriptional inhibition domain (181–388 amino acid residues) is rich in proline, indicating that KLF4 plays a dual role in activating and inhibiting gene transcription.^9^ In addition, KLF4 contains two independent nuclear localization signals: one located at the N‐terminal of the zinc finger structure and the other in front of the first zinc finger domain. These two nuclear localization signals are closely adjacent and can independently and effectively transfer KLF4 into the nucleus.^10^ KLF4 is a transcription factor that binds to its specific protein partner and is involved in the regulation of many cell biological processes, including proliferation, differentiation, and apoptosis.^11^ In addition to transcriptional regulation, KLF4 also performs posttranslational modifications through phosphorylation, ubiquitination, acetylation, threoninylation, and methylation. These modifications regulate KLF4 function by altering protein stability, DNA binding capacity, and transcriptional activity.^12^

Studies have shown that KLF4 has multiple roles, such as inhibiting and promoting tumor progression, regulating the cell cycle, influencing macrophage polarization, and influencing atherosclerosis.^13^, ^14^ Applicant's previous studies have found that KLF4 overexpression enhances gemcitabine sensitivity in pancreatic ductal adenocarcinoma by inhibiting ZEB1 expression.^15^ In addition, lipopolysaccharide (LPS)‐induced increased expression of KLF4 in microglia and astrocyte and KLF4 mediates neuroinflammation caused by the bacterial endotoxin LPS.^16^ KLF4 mediates LPS‐induced inflammatory injury and apoptosis in human lung fibroblasts.^17^ Qian Sun et al.^18^ found that oxidative stress upregulates KLF4 expression and activates the interleukin‐17 receptor (IL‐17A), thereby promoting IL‐1β and IL‐8, and then inducing inflammation and apoptosis of retinal pigment epithelium cells. The above studies indicate that KLF4 can be studied as a novel anti‐inflammatory target. KLF4 is rarely reported in UC. The study found that in a mouse model in which intestinal epithelial cells specifically knocked out KLF4, the expression of KLF4 was increased in the intestinal epithelial cells induced by DSS, and the symptoms of chronic colitis induced by DSS were reduced, suggesting that KLF4 may be involved in the occurrence and development of UC.^19^ However, the specific pathogenesis of KLF4 involvement in UC remains to be further explored.

## 2 MATERIALS AND METHODS

### 2.1 Clinical sample

Colon biopsy specimens from 30 outpatients and inpatients with UC admitted to the provincial hospital affiliated to Shandong First Medical University were collected (taken from the most obvious lesion (e.g., rectum, sigmoid colon), and the normal tissue adjacent to the colon cancer (more than 5 cm from the cancer tissue) surgically removed from 30 patients in the same period was taken as the control group, all of which were confirmed by pathological examination. Inclusion and exclusion criteria for UC patients: Inclusion criteria: ① The diagnostic criteria of UC comply with the criteria in the Consensus on Diagnosis and Treatment of Inflammatory bowel disease (2018 Beijing) issued by the Inflammatory bowel disease Group of the Digestive Branch of the Chinese Medical Association in 2018; ② Confirmed by colonoscopy and pathological biopsy. Exclusion criteria: ① Malignant tumor; ② Intestinal obstruction and perforation; ③ Severe bleeding and coagulation dysfunction; ④ Autoimmune diseases; ⑤ Other intestinal diseases. The sample collection process strictly complies with the provisions of the Human subject research (meeting the standards of the Ethics Committee and signing the informed consent of patients), and has been approved by the Ethics Committee. Patients were included in the study after signing the informed written consent.

### 2.2 Cell culture

Human colon epithelial cell line, NCM460, was purchased from Shanghai Hongshun Biological (Shanghai, China) and cultured in Eagle's Minimum Essential Medium supplemented with 10% FBS (Invitrogen), 2 mM l‐glutamine, 100 IU penicillin, and 100 mg/mL streptomycin at 37°C in a humidified incubator containing 5% CO_2_.

### 2.3 Immunohistochemical analysis

The immunohistochemical method of Pandurangan et al. was adopted with some modifications.^20^ Paraffin‐embedded tissue sections of 5 µm thickness were rehydrated in xylene and then in graded ethanol solutions. Then, the slides were incubated with HistoVT (10×, pH 7.0) (Nacalai Tesque) antigen retrieval solution for 20 min at 90°C and then cooled to room temperature. The slides were then blocked with 5% bovine serum albumin (BSA) in TBS‐Tween 20 (TBST) for 2 h. The sections were then incubated with primary antibody overnight at 4°C for immunostaining. The primary antibody: KLF4 (1:100; Proteintech, 11880‐1‐AP, China), TXNIP (1:100; Proteintech, 18243‐1‐AP, China), NLRP3 (1:100; Proteintech, 27458‐1‐AP, China), Caspase‐1 (1:100; Proteintech, 22915‐1‐AP, China), GSDMD (1:100; Proteintech, 20770‐1‐AP, China). After washing the slides thrice with TBST, the sections were incubated with the appropriate secondary antibodies in TBST with 5% BSA for 2 h at room temperature. The sections were then washed with TBST and incubated for 5 min with a peroxidase stain from a peroxidase stain DAB kit following the instructions provided by the manufacturer (Nacalai Tesque). Counterstaining was performed using hematoxylin (Cell Path), and the slides were photographed under a light microscope (Nikon ECLIPSE 80i; Nikon Corporation).

### 2.4 Western blot analysis

Immunoblot analysis was performed according to the method of Ashokkumar and Sudhandiran,^21^ with some modifications. Briefly, colonic tissues were removed and washed in ice‐cold PBS. The whole colonic tissue was cut into pieces and homogenized in five volumes of ice‐cold homogenizing buffer (0.1 mM NaCl, 0.1 M Tris‐HCl, 0.001 M EDTA, 1 mM PMSF, 1 mg/mL aprotonin, and 0.1 mM leupeptin) and centrifuged at 3000*g* for 1 h at 4°C. The protein contents of the supernatants were estimated using BSA as the standard. The extracts were heated in a boiling water bath for 5 min, and 40 µg protein samples were subjected to 10% sodium dodecyl sulfatepolyacrylamide gel electrophoresis and transferred to nitrocellulose membrane (Millipore Corp, HATF00010) using a transfer apparatus (Bio‐Rad) at room temperature. The membranes were blocked with 5% skimmed milk in TBST at room temperature and subsequently incubated overnight with primary antibody at 4°C. The following primary antibodies were used: KLF4 (1:1000; Proteintech, 11880‐1‐AP, China), TXNIP (1:1000; Proteintech, 18243‐1‐AP, China), NLRP3 (1:1000; Proteintech, 27458‐1‐AP, China), Caspase‐1 (1:1000; Proteintech, 22915‐1‐AP, China), GSDMD (1:1000; Proteintech, 20770‐1‐AP, China), β‐actin (1:2000; Proteintech, 66009‐1‐Ig, China). They were then incubated with the corresponding horseradish peroxidase‐conjugated secondary antibody (Santa Cruz Biotechnology Inc.) for 1 h at room temperature. The protein bands were visualized, and the quantification of the respective blots was performed using Image J software (NIH).

### 2.5 RNA isolation and quantitative polymerase chain reaction (qPCR)

Isolation of RNA from colonic tissue and real‐time PCR (RT‐PCR) were performed following the method described by Pandurangan et al.^22^ Total RNA from colonic tissue was extracted using a QIA shredder and RNeasy Kit (Qiagen NV) following the manufacturer's instructions. Quantitative RT‐PCR was performed using an Eppendorf PCR system with QuantiFast SYBR Green PCR Master Mix (Qiagen NV), primers (10 mM), and 1 µg cDNA in a 20 μL reaction mixture. All target cDNAs and the standard β‐actin cDNA were analyzed in duplicate in three independent RT‐PCR assays. Thermal cycling was initiated with an activation step at 95°C for 30 s, followed by 40 cycles of 95°C for 5 s and 60°C for 30 s. Immediately after amplification, melt curves were generated to ensure the primer dimers and other nonspecific products had been minimized. The relative expressions of the target genes were analyzed by the $2^{‐∆∆C_{t}}$ method. The primer sequence was shown: *Klf4*: Forward primer: 5′‐ACCTACACAAAGAGTTCC CATC−3′, Reverse primer: 5′‐TGTGTTTACGGTAGTGCCTG−3′; *Txnip*: forward primer:5′‐TGTGTGAAGTTACTCGTGTCAAA−3′, reverse primer: 5′‐GCAGGTACT CCGAAGTCTGT−3′; *Nlrp3*: forward primer: 5′‐GGGACCCAGGGATGAGAGTGTT −3′, reverse primer: 5′‐TGCTGCTGAGGACCAAGGAGAT−3′; *Gsdmd*: forward primer: 5′‐TGGACCCTAACACCTGGCAGAC−3′, reverse primer: 5′‐AGCACCTCA GTCACCACGTACA‐3′; *Caspase‐1*: forward primer: 5′‐AGACCTCTGACAGCAC GTTCCT‐3′, reverse primer: 5′‐TCCCACAAATGCCTTCCCGAAT‐3′; *Asc*: forward primer: 5′‐AGTGGCTGCTGGATGCTCTGT‐3′, reverse primer: 5′‐GCACTGCCTGG TACTGCTCATC‐‐3′; β‐actin: Forward primer: 5′‐CACCTTCTACAATGAGCTGCG TGTG‐3′, 5′‐ ATAGCACAGCCTGGAT AGCAACGTAC‐3′.

### 2.6 Inflammasome stimulation and determination of pyroptotic cell death

For NLRP3 inflammasome stimulation, cells transfected with siRNA were stimulated by 200 ng/mL LPS (Sigma‐Aldrich) and 5 mM adenosine 5‐triphosphate (ATP) (SunShine Biotechnology). The morphology of pyrolytic cells was examined under a light microscopy. Cells were stained with propidium iodide to mark the membrane pores (Life Technology).

### 2.7 Enzyme‐linked immunosorbent assay (ELISA)

IL‐1β and IL‐18 levels were quantified by human IL‐1β ELISA Kit (P5106, Beyotime Biotechnology) and LIL‐18 ELISA Kit (PI558, Beyotime Biotechnology), respectively.

### 2.8 Dual‐luciferase

To detect the luciferase activity of Fluc and Rluc, cells were seeded in 24‐well plates in a 5% CO_2_ incubator. Next day, the promoters of TXNIP were constructed into the pGL3‐basic vector and these plasmids were transfected into NCM460 cells, treated them with LPS/ATP. Then the cells were harvested and lysed in cell lysis buffer (TransGen Biotech). Finally, the luciferase activity was measured with a Promega Dual‐Luciferases Reporter Assay kit (Promega E1980) according to the manufacturer's protocols after transfection. Relative Renilla luciferase activity was normalized to firefly luciferase activity.

### 2.9 Immunoprecipitation

HEK293 cells (ATCC, CRT‐1573) were cultured in DMEM with 10% FBS and 1% penicillin and streptomycin at 37°C in a humidified 5% CO_2_ atmosphere. The cells were transfected with TXNIP plasmid, and lysates were immunoprecipitated with a TXNIP antibody. Coimmunoprecipitated KLF4 was detected with an anti‐KLF4 antibody.

### 2.10 Lentivirus packaging and infection

To generate the lentiviral shRNA constructs against human KLF4, the target sequences were cloned into pLKO.1‐puro vector. The shRNA sequences are listed: shRNA1: 5′‐TTGGTGAGTCTTGGTTCTAA−3′; shRNA2: 5′‐CAGAACAAATGTG TTTTTCT‐3′. These pLKO.1 plasmids were transfected into human intestinal epithelial cells. RT‐qPCR and western blot assay identified this efficiency.

### 2.11 Statistical analysis

Data are expressed as means ± standard deviation (SD). Student's *t* test was used to compare values between two groups. Values obtained from three or more groups were compared using one‐way analysis of variance followed by Tukey's post hoc test. A *p* < .05 was considered significant.

## 3 RESULTS

### 3.1 Expression of KLF4, TXNIP, NLRP3 inflammatory body activation markers and pyroptosis markers in colon tissue of UC patients

To study the effect of KLF4 and TXNIP on pyroptosis of intestinal epithelium in UC. We detected the mRNA expression and protein level of KLF4, TXNIP, and key factors of pyroptosis (NLRP3, Caspase‐1, and GSDMD) in the diseased colon tissue of patients diagnosed with active UC and in the colon tissue of the control group. The quantitative analysis results of qRT‐PCR showed that compared with the control group, the mRNA expression of KLF4 (Figure 1A), TXNIP (Figure 1B), NLRP3 (Figure 1C), Caspase‐1 (Figure 1D), and GSDMD (Figure 1E) in 15 cases of UC tissue was significantly increased. The immunohistochemical results showed that compared with the control group, the protein expression of KLF4, TXNIP, NLRP3, Cleaved Caspase‐1, and GSDMD in UC colon mucosa tissue increased, mainly distributed in intestinal epithelial cells (Figure 1F). Western blot results showed that compared with the control group, the protein expression of KLF4, TXNIP, NLRP3, Cleared caspase‐1, and GSDMD in UC colon mucosa tissue increased (Figure 1G). These results indicate that the expression levels of KLF4, TXNIP, and key factors of pyroptosis (NLRP3, Caspase‐1, and GSDMD) in colonic tissue of patients with UC are increased and mainly distributed in intestinal epithelial cells.

**Figure 1:**
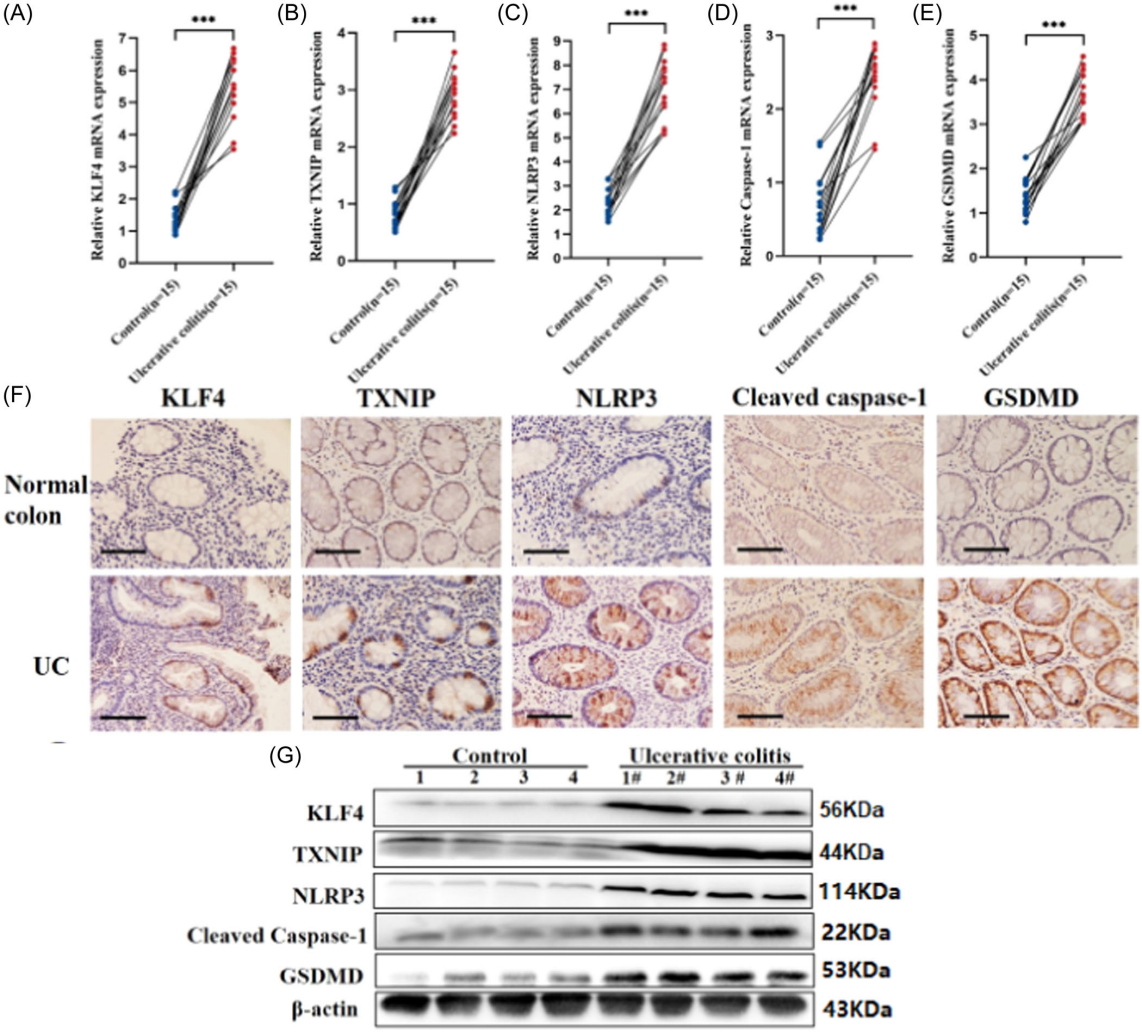
Expression of KLF4, TXNIP, NLRP3 inflammatory body activation markers and pyroptosis markers in colon tissue of ulcerative colitis (UC) patients. (A‐E) Quantitative reverse transcription polymerase chain reaction assay showed that KLF4, TXNIP, NLRP3, Caspase‐1, and gasdermin D (GSDMD) mRNA expression were elevated in UC tissues. *N* = 15. (F) Immunohistochemical assay exhibited that KLF4, TXNIP, NLRP3, Caspase‐1, and GSDMD expression were elevated in UC tissues. *N* = 4. (G) Western blot assay identified that the protein of KLF4, TXNIP, NLRP3, Caspase‐1, and GSDMD expression were elevated in UC tissues. *N* = 4. ***p < .001.

### 3.2 Expression of KLF4 and TXNIP in LPS/ATP‐induced intestinal pyroptosis model

To study the effect of KLF4 and TXNIP on intestinal pyroptosis. We cultured human intestinal epithelial cell line (NCM460) in vitro. The human intestinal epithelial cell line was stimulated with 200 ng/mL of LPS, and then incubated with 5 mM of 5‐neneneba adenosine triphosphate (ATP) for 24 and 48 h. Western blot was used to detect the expression levels of KLF4, TXNIP, NLRP3, Cleave‐Caspase‐1, and GSDMD in NCM460 cells (Figure 2A,B). By collecting cell culture supernatant and analyzing the concentration of IL‐1β and IL‐18 using an ELISA detection kit. We found that after LPS/ATP combined stimulation, the expression levels of KLF4, TXNIP, NLRP3, Cleave‐Caspase‐1, and GSDMD were significantly increased (Figure 2A,B). In addition, we also found that the IL‐1β and IL‐18 concentration in the supernatant were increased (Figure 2C,D).

**Figure 2:**
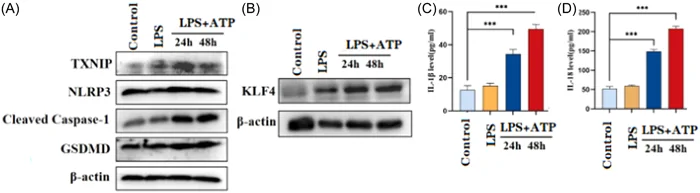
Expression levels of KLF4, TXNIP, and key factors of pyroptosis and release of inflammatory factors in LPS/ATP‐induced intestinal pyroptosis model. (A) Western blot assay identified that TXNIP, NLRP3, Cleaved Caspase‐1, GSDMD expression were significantly upregulation in lipopolysaccharide (LPS)/adenosine 5‐triphosphate (ATP)‐induced intestinal pyroptosis model. (B) After stimulation for 24 and 48 h, western blot assay identified that KLF4 expression was significantly upregulation in LPS/ATP‐induced intestinal pyroptosis model. (C and D) After stimulation for 24 and 48 h, enzyme‐linked immunosorbent assay identified that interleukin (IL)‐1β and IL‐18 levels were significantly elevated in LPS/ATP‐induced intestinal pyroptosis model. ***p < .001.

### 3.3 The regulatory effect of KLF4 on TXNIP transcription

We apply the online promoter analysis software UCSC (http://genome.ucsc.edu/) predict the possible transcription factors in the TXNIP gene promoter region, and then use the JASPAR transcription factor database (http://jaspar.genereg.net/) analysis revealed the presence of five KLF4 binding sites in the promoter region of TXNIP (with a relative profile score threshold of 85%) (Figure 3A). Construct Luciferase reporter gene vector, which contains KLF4 binding site or mutant binding site (Figure 3B). The experimental results of luciferase report show that KLF4 positively regulates the TXNIP promoter‐mediated luciferase activity (Figure 3C). At the same time, we found a direct interaction between KLF4 and TXNIP through yeast hybridization (Figure 3D). Because we have constructed plasmids for KLF4‐AD and TXNIP‐BD respectively, yeast can only survive on the SD‐Leu‐Trp‐His medium when the AD and BD of the two plasmids interact, otherwise they cannot survive. Co‐IP experiment also demonstrated similiar result (Figure 3E). The above results indicate that KLF4 positively regulates the transcriptional expression of TXNIP, and KLF4 may be the upstream regulator of TXNIP participating in UC.

**Figure 3:**
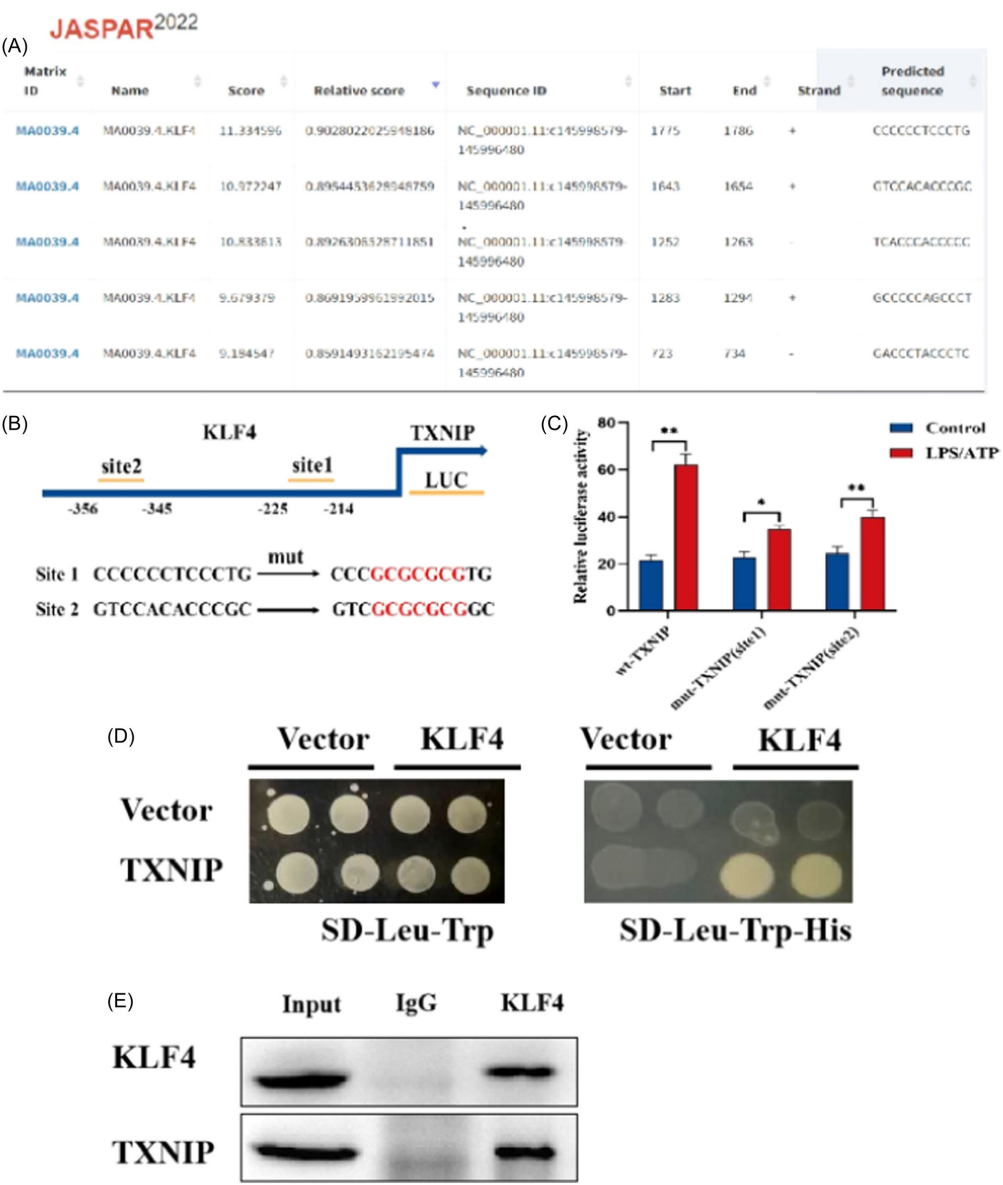
KLF4 positive regulation of TXNIP transcription expression. (A) Using UCSC online promoter analysis software to predict the promoter region of TXNIP gene and the transcription factor database JASPAR for transcription factors that may interact with the promoter region of TXNIP. We found that there are five KLF4 binding sites in the promoter region of TXNIP. (B) Schematic diagram of binding site primer design. (C) Luciferase report confirms that KLF4 regulates TXNIP transcription in lipopolysaccharide/adenosine 5‐triphosphate‐induced intestinal pyroptosis model. (D) Y2H screening for interactions between KLF4 and TXNIP. We have constructed plasmids for KLF4‐AD and TXNIP‐BD, respectively, yeast can only survive on the SD‐Leu‐Trp‐ His medium when the AD and BD of the two plasmids interact, otherwise they cannot survive. (E) CO‐IP assay showed that there is a direct interaction between KLF4 and TXNIP. *p < .5; **p < .01.

### 3.4 Detection of KLF4 and TXNIP protein expression in human intestinal epithelial cells silenced with KLF4

The research team designed and synthesized a shRNA interference sequence targeting KLF4, amplified it by PCR and connected it to the pLKO.1‐Uro lentivirus interference plasmid (abbreviated as pLKO.1) to obtain a recombinant lentivirus plasmid pLKO.1‐shKLF4. Packaging was carried out in 293T cells to produce recombinant lentivirus particles, which infected intestinal epithelial cell lines and produced human intestinal epithelial cell lines KLF4 shRNA 1‐NCM460 and KLF4 shRNA 2‐NCM460 that silenced KLF4. The cell lines were validated by quantitative reverse transcription PCR and western blot experiments (Figure 4A,B). The results indicate the successful construction of a stable silencing KLF4 human intestinal epithelial cell line. After stimulation of intestinal epithelial cells with ATP/LPS, it was found that the mRNA expression of TXNIP (Figure 4C), NLRP3 (Figure 4D), ASC (Figure 4E), Caspase‐1 (Figure 4F), and GSDMD (Figure 4G) was significantly lower than that of intestinal epithelial cells treated with LPS/ATP, with inflammatory factor 1L‐1β The levels of IL‐18 (Figure 4H) and IL‐18 (Figure 4I) were also significantly lower than those of intestinal epithelial cells treated with LPS/ATP, indicating that TXNIP/NLRP3 mediated pyroptosis in UC intestinal epithelial cells.

**Figure 4:**
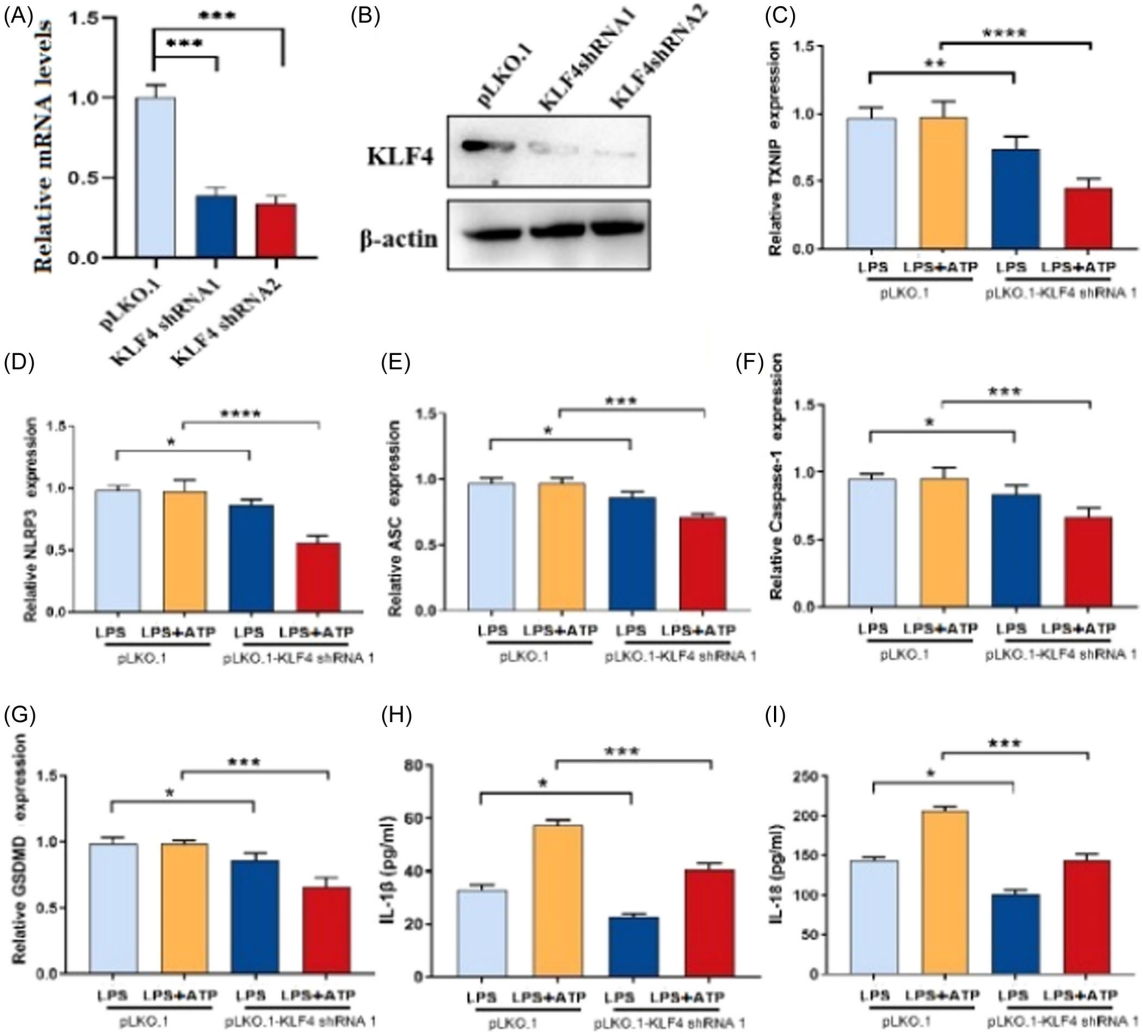
Construction of a human intestinal epithelial cell line silencing KLF4 and detection of protein expressions. (A) The transcription level of KLF4 was significantly downregulated in KLF4‐silenced human intestinal epithelial cells. (B) Western blot identified that KLF4 protein was significantly downregulated in KLF4‐silenced human intestinal epithelial cells. (C–G) QPCR assay showed that TXNIP (C), NLRP3 (D), ASC (E), Caspase‐1 (F), and gasdermin D (G) levels were significantly decreased in lipopolysaccharide (LPS)/adenosine 5‐triphosphate (ATP)‐induced intestinal pyroptosis model. (H) Interleukin (IL)‐1β level was remarkably decreased in LPS/ATP‐induced intestinal pyroptosis model. (I) IL‐18 level was remarkably decreased in LPS/ATP‐induced intestinal pyroptosis model. *p < .5; ***p < .001.

## 4 DISCUSSION

UC is an inflammatory bowel disease characterized by imbalance microbiota, abnormal activation of innate immunity, and the interaction between these factors.^23^, ^24^ It is widely believed that NLRP3‐like receptors, as a type of innate immune receptor,^25^, ^26^ can trigger immune and inflammatory responses after infection. NLRP3 inflammasome is a representative molecule of this family. In UC animal models (induced by DSS), the expression of NLRP3 increases,^27^ but the lack of NLRP3 is not sensitive to DSS models.^28^ Initiating NLRP3 depends on transcription activation of NF‐κB.

Cell death includes programmed death and necrosis, and the discovery of a new type of cell death in programmed death—cell pyroptosis. Cell pyroptosis is a type of proinflammatory programmed cell death that mainly relies on Caspase, characterized by rapid membrane rupture and the release of proinflammatory cell contents.^5^

With the deepening of the research on the pathogenesis of UC, the current research has shown that intestinal pyroptosis is involved in the occurrence and development of UC. Research has confirmed that in UC patients and the mouse UC model induced by dextran sulfate sodium (DSS), the key effectors of pyroptosis, GSDMB and GSDMD, are increased in expression and located in intestinal epithelial cells. GSDMD deficiency can reduce intestinal inflammation in experimental UC.^29^, ^30^ The mRNA expression and protein expression of pyroptosis‐related factors NLRP3, Caspase‐1, and GSDMD increased in colon tissue samples from UC patients and DSS‐induced UC mice.^31^ Assembly and activation of NLRP3, ASC, pro‐caspase‐1 inflammasomes in colon epithelial cells, as well as increased IL‐1β and IL‐18, are associated with the onset and progression of UC.^32^, ^33^ In this study, these indicators were consistent with our results.

Under physiological conditions, KLF4 is mainly expressed in the postmitotic differentiated epithelial cells of the small intestine and colon.^34^, ^35^ Klf4 is critical in regulating intestinal epithelial cell homeostasis in vivo.^36^ In our study, we apply the online promoter analysis software UCSC (http://genome.ucsc. edu/) to predict the possible transcription factors in the TXNIP gene promoter region. Then using the JASPAR transcription factor database (http://jaspar.genereg.net/) to analyze. Analysis revealed the presence of five KLF4 binding sites in the promoter region of TXNIP (with a relative profile score threshold of 85%). Luciferase Reporter gene vectors containing KLF4 binding sites or mutant binding sites were constructed. However, a recent study showed that KLF4 was enriched on the NLRP3 promoter and improved NLRP3 expression. NLRP3 overexpression reversed the inhibition of sh‐KLF4 on pyroptosis of NEpCs in AR mice.^37^ Overall, our results demonstrated that KLF4 positively regulates the TXNIP promoter‐mediated luciferase activity.

According to the content of this study, we concluded that: transcription factor KLF4 upregulates the expression of TXNIP, activates NLRP3 inflammatory bodies, and then activates Caspase‐1 mediated intestinal Pyroptosis and inflammatory factor release, leading to the occurrence and development of UC (Figure 5).

**Figure 5:**
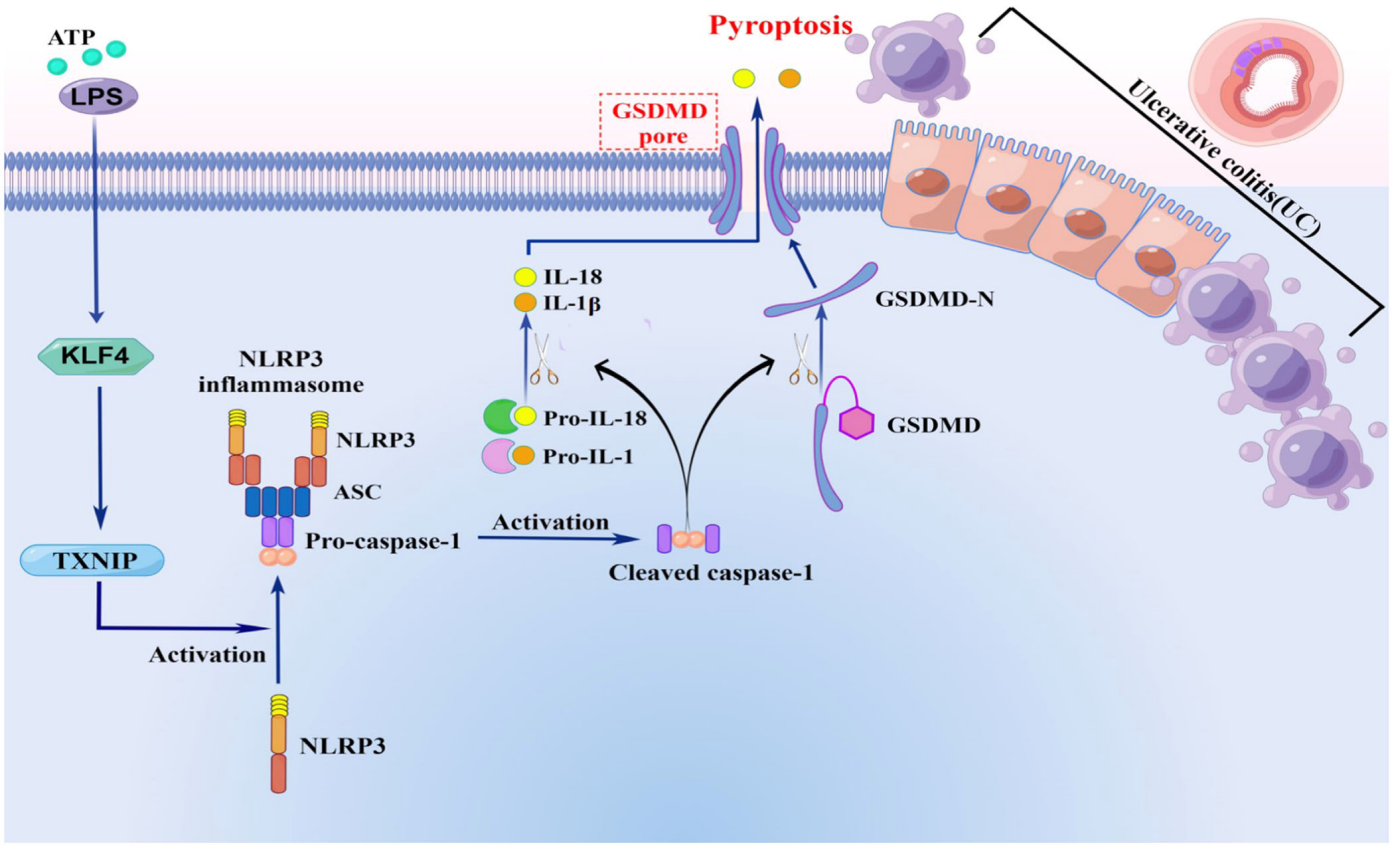
KLF participates in the pyroptosis process of ulcerative enteritis through positive regulation of TXNIP/NLRP3 pathway. Adenosine 5‐triphosphate/lipopolysaccharide stimulation can activate the expression of KLF4, and KLF4 is regulating the expression of TXNIP. At the same time, TXNIP/NLRP3 signaling pathway is the classic pathway regulating pyroptosis. Therefore, in this study, KLF4 regulates TXNIP/NLRP3 pathway to participate in the pyroptosis process of Ulcerative colitis.

In conclusion, KLF4 affects the pyroptosis process of UC by positively regulating the expression of TNXIP, which provides a new therapeutic strategy for the treatment of UC.

There are also some limitations to our research; for example, (1) we need to construct an animal model to confirm the results of our in vitro experiments through in vivo research. (2) We also need to knock out KLF4 or TXNIP separately to detect relevant indicator changes.

## AUTHOR CONTRIBUTIONS

Yuan Chen and Zhiyi Wang designed the study and supervised the data collection. Yuan Chen, Lifeng Sun, Haiyan Liu, Jiamei Li, and Lu Guo analyzed the data and interpreted the data. Zhiyi Wang prepared the manuscript for publication and reviewed the draft of the manuscript. All authors have read and approved the manuscript.

## CONFLICT OF INTEREST STATEMENT

The authors declare no conflict of interest.

## Data Availability

We will provide data under reasonable demand.
