# EXPRESSION OF CONCERN: KLF4 Interacts With TXNIP to Modulate the Pyroptosis in Ulcerative Colitis via Regulating NLRP3 Signaling

**DOI:** 10.1002/iid3.70503

**Published:** 2026-09-10

**EXPRESSION OF CONCERN**: Y. Chen, L. Sun, H. Liu, J. Li, L. Guo, and Z. Wang, “KLF4 Interacts With TXNIP to Modulate the Pyroptosis in Ulcerative Colitis via Regulating NLRP3 Signaling,” *Immunity, Inflammation and Disease* 12, no. 2 (2024): e1199, https://doi.org/10.1002/iid3.1199.

This Expression of Concern is for the above article, published online on 27 February 2024 in Wiley Online Library (https://onlinelibrary.wiley.com), and has been issued by agreement between the journal Editor‐in‐Chief, Alexander Hutchison; and John Wiley & Sons, Ltd. The Expression of Concern has been agreed following an investigation which identified concerns regarding the ethics approval and participant consent for the study. Although the article states that ethical approval was obtained, it does not identify the approving ethics committee or provide an approval reference number. The authors were contacted and asked to provide clarification and supporting information but did not respond. As the journal has been unable to verify the ethics approval and consent information provided in the article, the editors have decided to issue an Expression of Concern to alert readers to these concerns.

